# Evaluation of the Aspartate Aminotransferase to Platelet Ratio Index for Predicting In-Hospital Mortality in Cardiogenic Shock Patients Admitted to the Intensive Care Unit

**DOI:** 10.31083/RCM26590

**Published:** 2025-04-16

**Authors:** Min Yang, Dandan Liu, Yu Liu

**Affiliations:** ^1^Department of General Disease, The Eighth Affiliated Hospital of Sun Yat-sen University, 51800 Shenzhen, Guangdong, China; ^2^Department of Neurosurgery, Zhongshan Hospital of Traditional Chinese Medicine, 528400 Zhongshan, Guangdong, China

**Keywords:** aspartate aminotransferase to platelet ratio index, cardiogenic shock, intensive care unit, predict

## Abstract

**Backgrounds:**

This study aimed to investigate the conceivable utility of the aspartate aminotransferase to platelet ratio index (APRI) in prognostic prediction for patients with cardiogenic shock (CS) hospitalized in the intensive care unit (ICU).

**Methods:**

Data for patients diagnosed with CS were obtained from the Medical Information Mart for Intensive Care-IV (MIMIC-IV) database and categorized into groups based on the APRI quartiles. The primary endpoint encompassed in-hospital and ICU mortality rates. The secondary outcomes included sepsis and acute kidney injury (AKI). Kaplan–Meier survival analysis was utilized to assess differences in main endpoints among groups categorized by their APRI.

**Results:**

This study collected data from 1808 patients diagnosed with CS. Multivariate Cox regression analysis indicated that an elevated APRI was independently correlated with a heightened risk of in-hospital mortality (hazard ratio (HR) 1.005 [95% confidence interval (CI) 1.003–1.007]; *p* < 0.001) and ICU mortality (HR 1.005 [95% CI 1.003–1.007]; *p* < 0.001). Multivariate logistic regression analysis demonstrated that APRI was independently correlated with a heightened risk of sepsis (odds ratio (OR) 1.106 [95% CI 1.070–1.144]; *p* < 0.001) and AKI (OR 1.054 [95% CI 1.035–1.073]; *p* < 0.001).

**Conclusions:**

An increased APRI was linked to worse clinical outcomes in critically ill patients with cirrhosis. Nevertheless, further extensive prospective investigations are needed to validate these findings.

## 1. Introduction

Cardiogenic shock (CS) frequently occurs in intensive care units (ICUs) and is linked to a significantly elevated death rate. Indeed, the short-term mortality rate surpasses 50%, with the risk of death within 30 days being substantially greater [1, 2]. Recent studies have established that interventions beyond culprit vessel revascularization for patients with acute myocardial infarction (MI) do not significantly enhance the short-term survival rate of those with CS and that no effective treatments exist for patients with non-MI causes of CS, thereby presenting informative encouragement for clinicians [3, 4, 5]. Consequently, it is necessary to investigate readily applicable biomarkers to enhance mortality prediction in patients diagnosed with CS and admitted to the ICU to aid doctors in formulating customized management plans.

Recently, the aspartate aminotransferase to platelet ratio index (APRI), derived by splitting the serum aspartate aminotransferase (AST) level by the serum platelet (PLT) level, has emerged as a user-friendly and precise biomarker, regarded as a non-invasive screening tool for liver fibrosis and non-alcoholic fatty liver disease [6, 7, 8]. Numerous studies have validated that APRI possesses significant prognostic value in the prognosis of many diseases, including liver cancer [9] and colorectal cancer [10]. However, the predictive function of APRI in CS patients remains unclear. Therefore, this study uses the Medical Information Mart for Intensive Care-IV (MIMIC-IV) database to ascertain the utility of the APRI in prognostic prediction for patients with CS admitted to the ICU. This retrospective observational study represents the inaugural investigation employing the liver fibrosis indicator APRI to forecast outcomes in critically ill CS patients.

## 2. Materials and Methods

### 2.1 Data Source

The current retrospective observational study utilized health-related data obtained from the MIMIC-IV database. This database comprises statistics on patients in the ICU of the Beth Israel Deaconess Medical Center (BIDMC), a major tertiary hospital in Boston, USA, from 2008 to 2019. The BIDMC Institutional Review Board exempted the study from the informed consent requirement and permitted the dissemination of research resources, guaranteeing that all data were anonymized. One of the writers (LY) satisfied all requisite criteria for database access and secured the necessary permissions.

### 2.2 Cohort Selection

**Fig. 1.**
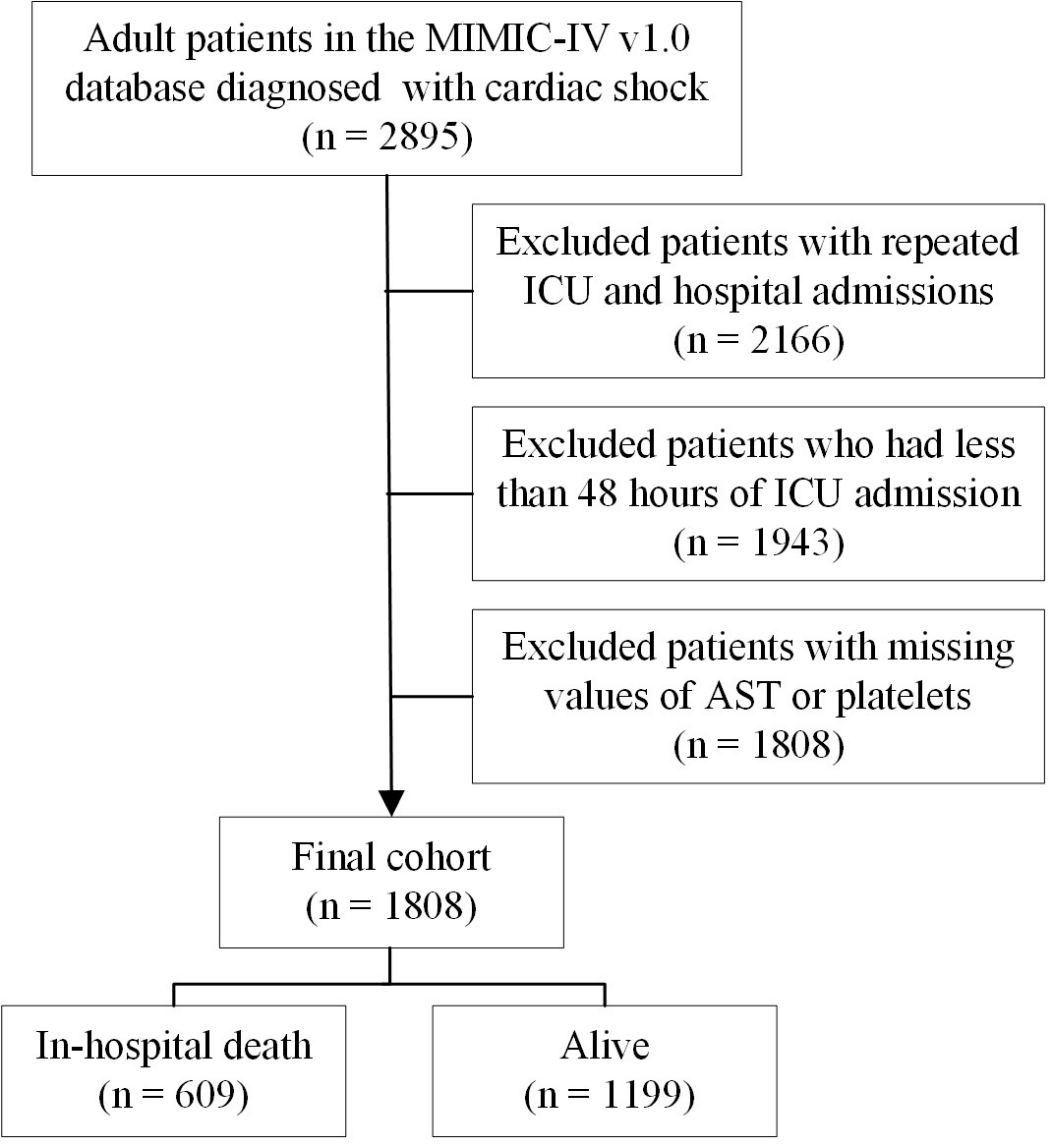
**A flowchart of this study**. MIMIC-IV, Medical Information Mart for Intensive Care-IV; ICU, intensive care unit; AST, aspartate aminotransferase.

CS was delineated based on the Ninth Revision of the International Classification of Diseases (ICD-9) codes. CS was characterized by a systolic blood pressure of 90 mmHg and indicators of hypoperfusion, including altered mental status or disorientation, cold extremities, and oliguria of 2 mmol/L [11]. The exclusion criteria included multiple ICU and hospital admissions, hospital stays shorter than 48 hours, and incomplete data for either AST or platelets. In total, 1808 patients with CS were analyzed, comprising 609 cases of in-hospital mortality and 1199 survivors (Fig. 1).

### 2.3 Data Collection

Initial information was obtained from the MIMIC-IV database through Structured Query Language (SQL) in PostgreSQL (version 9.6, PostgreSQL Global Development Group, Berkeley, CA, USA). This included data on age, gender, weight, severity scores, comorbidities, vital signs, and laboratory tests. However, we can only include the initial measurements of laboratory tests taken within 48 hours of ICU admission because of database constraints, and we cannot dynamically retrieve laboratory test information. Comorbidities included congestive heart failure [12], atrial fibrillation [13], hypertension [14], diabetes [15], chronic kidney disease [16], and chronic obstructive pulmonary disease (COPD) [17].

### 2.4 Endpoints

The main focus included encompassing mortality rates during hospitalization and in the ICU. The secondary endpoints included sepsis and acute kidney injury (AKI). Patients were identified with sepsis according to the sepsis 3.0 criteria [18]. AKI was defined based on the Kidney Disease: Improving Global Outcomes (KDIGO)-AKI criteria [19].

### 2.5 Statistical Analysis

Statistical analyses were conducted utilizing SPSS 22.0 (IBM Corp., Armonk, NY, USA), X-tile, and R software version 4.1.2 (R Foundation for Statistical Computing, Vienna, Austria). To enhance the prognostic value of the APRI in predicting clinical outcomes for critically ill patients with CS, the study participants were categorized into four groups based on the index quartiles. Based on the data characteristics, continuous variables are presented as the mean ± standard deviation or median (interquartile range); meanwhile, categorical variables were assessed using Chi-square tests or Fisher’s exact test. The Kaplan–Meier survival analysis was employed to evaluate the incidence rate of primary endpoints across groups categorized by varying levels of the APRI, with comparisons made using the log-rank test.

Clinically pertinent and prognostic covariates were incorporated into the multivariate model: Model 1: unadjusted; Model 2: adjusted for age, gender, ethnicity, and comorbidity; Model 3: adjusted for age, gender, ethnicity, comorbidity, severity scores, and laboratory findings. A two-tailed *p*-value of less than 0.05 was used to indicate statistical significance.

## 3. Results

### 3.1 Patient Characteristics

Overall, this study included data from 1808 patients (Table 1). The patients were categorized into four groups based on the observed APRI quartiles: Q1, <0.39; Q2, 0.39–0.79; Q3, 0.8–2.62; and Q4, >2.62. Compared to the lowest APRI quartile, patients in the highest APRI quartile exhibited a younger demographic, a greater male proportion, and a reduced prevalence of congestive heart failure, chronic kidney disease, and COPD. These patients also demonstrated a higher Charlson comorbidity index, elevated severity scores for Sequential Organ Failure Assessment (SOFA), Oxford Acute Severity of Illness Score (OASIS), and Acute Physiology Score III (APS-III), increased mean arterial pressure, respiratory rate, and elevated levels of several laboratory results (all *p* < 0.05) (Table 1). Furthermore, in comparison to the lowest APRI quartile, patients in the highest APRI quartile exhibited a greater incidence of in-hospital mortality, ICU mortality, sepsis, and acute kidney injury (all *p* < 0.05) (Table 1).

**Table 1.** **Comparisons of baseline characteristics for all patients**.

Characteristics		Overall	Q1 (<0.39)	Q2 (0.39–0.79)	Q3 (0.80–2.62)	Q4 (>2.62)	*p*-value
N		1808	456	450	451	451	
Age, years old		69.7 ± 14.2	69.7 ± 13.8	71.8 ± 13.2	69.6 ± 14.1	67.8 ± 14.5	<0.001
Gender, male, n (%)		1099 (60.8)	253 (55.5)	269 (59.8)	277 (61.4)	300 (66.5)	0.008
Ethnicity, n (%)							0.006
	White	1176 (65.0)	305 (66.9)	320 (71.1)	281 (62.3)	270 (59.9)	
	Black	232 (12.8)	63 (13.8)	50 (11.1)	59 (13.1)	60 (13.3)	
	Others	400 (22.2)	88 (19.3)	80 (17.8)	111 (24.6)	121 (26.8)	
Comorbidity, n (%)							
	Congestive heart failure	1464 (81.0)	380 (83.3)	380 (84.4)	359 (79.6)	345 (76.5)	0.009
	Atrial fibrillation	993 (54.9)	255 (55.9)	263 (58.4)	246 (54.5)	229 (50.8)	0.133
	Hypertension	708 (39.2)	180 (39.5)	171 (38.0)	184 (40.8)	173 (38.4)	0.825
	Diabetes	705 (39.0)	198 (43.4)	174 (38.7)	171 (37.9)	162 (35.9)	0.122
	Chronic kidney disease	728 (40.3)	199 (43.6)	211 (46.9)	174 (38.6)	144 (31.9)	<0.001
	COPD	565 (31.3)	165 (36.2)	147 (32.7)	121 (26.8)	132 (29.3)	0.015
Charlson index, points		7.0 (5.0, 9.0)	7.0 (5.0, 9.0)	7.0 (6.0, 9.0)	7.0 (6.0, 9.0)	7.0 (6.0, 10.0)	0.001
Severity scores, points							
	SOFA	9.0 (6.0, 12.0)	7.0 (4.0, 10.0)	8.0 (5.0, 11.0)	9.0 (6.0, 14.0)	10.0 (7.0, 13.0)	<0.001
	OASIS	37.4 ± 10.0	35.5 ± 9.8	36.2 ± 9.6	37.6 ± 9.9	40.5 ± 10.0	<0.001
	APS-III	63.0 (46.0, 85.0)	56.0 (43.0, 76.0)	59.0 (44.0, 79.0)	62.0 (45.0, 85.0)	71.0 (55.0, 96.0)	<0.001
Vital signs							
	MAP, mmHg	78.9 ± 19.1	77.6 ± 18.4	76.7 ± 16.3	80.3 ± 21.6	81.2 ± 19.5	0.001
	Heart rate, bpm	91.9 ± 21.6	92.5 ± 21.5	90.9 ± 21.4	91.7 ± 21.9	92.4 ± 21.6	0.695
	RR, bpm	20.9 ± 6.6	20.1 ± 6.2	20.9 ± 6.7	21.1 ± 6.8	21.4 ± 6.4	0.020
	SpO_2_, %	96.0 ± 5.1	95.9 ± 5.1	96.3 ± 4.1	95.9 ± 5.6	95.8 ± 5.5	0.485
Laboratory values							
	WBC, ×10^9^/L	11.2 (7.9, 15.5)	10.1 (7.6, 13.6)	9.8 (7.2, 13.7)	11.7 (8.1, 16.8)	12.7 (9.1, 18.0)	<0.001
	Hemoglobin, g/dL	11.3 ± 2.4	11.2 ± 2.2	11.2 ± 2.3	11.2 ± 2.4	11.6 ± 2.6	0.019
	Platelet, ×10^9^/L	198 (150, 262)	246 (196, 308)	189 (148, 308)	194 (140, 262)	172 (120, 230)	<0.001
	AST, U/L	60 (29, 191)	23 (18, 37)	40 (30, 52)	102 (70, 232)	472 (257, 1089)	<0.001
	ALT, U/L	38 (19, 113)	18 (23, 26)	26 (18, 40)	57 (33, 103)	284 (113, 751)	<0.001
	APRI	5.4 ± 1.1	0.25 ± 0.08	0.55 ± 0.11	1.46 ± 0.55	19.53 ± 4.5	<0.001
	Albumin, g/dL	3.3 ± 0.7	3.4 ± 0.7	3.4 ± 0.7	3.2 ± 0.7	3.2 ± 0.7	<0.001
	Anion gap, mEq/L	17.2 ± 4.7	16.0 ± 3.3	16.6 ± 4.4	17.2 ± 4.6	18.9 ± 5.7	<0.001
	Bicarbonate, mEq/L	22.1 ± 5.0	24.1 ± 4.4	23.1 ± 5.1	21.3 ± 4.4	19.7 ± 5.1	<0.001
	Glucose, mg/dL	143 (109, 198)	129 (104, 168)	138 (108, 184)	154 (113, 218)	156 (118, 235)	<0.001
	BUN, mg/dL	31 (20, 49)	30 (19, 48)	33 (21, 51)	29 (19, 48)	31 (20, 50)	0.129
	Creatinine, mg/dL	1.5 (1.0, 2.2)	1.3 (1.0, 2.1)	1.4 (1.0, 2.2)	1.5 (1.0, 2.2)	1.6 (1.1, 2.3)	0.012
	Potassium, mmol/L	4.5 ± 0.9	4.4 ± 0.8	4.4 ± 0.8	4.6 ± 1.0	4.6 ± 1.0	<0.001
	Sodium, mmol/L	137.3 ± 5.5	137.5 ± 5.2	137.2 ± 5.2	137.2 ± 5.5	137.3 ± 5.9	0.817
	PT, s	15.1 (13.0, 21.0)	14.3 (12.5, 17.7)	15.0 (13.1, 20.4)	15.0 (13.0, 21.2)	17.1 (13.5, 24.2)	<0.001
	APTT, s	34.8 (29.1, 49.7)	32.1 (27.9, 40.6)	34.0 (28.6, 44.8)	37.3 (29.8, 57.5)	38.0 (30.5, 67.5)	<0.001
	INR	1.4 (1.2, 1.9)	1.3 (1.1, 1.6)	1.4 (1.2, 2.0)	1.4 (1.2, 2.1)	1.6 (1.2, 2.2)	<0.001
Primary outcomes							
	Hospital LOS, days	11.9 (6.8, 19.7)	13.5 (8.0, 22.1)	12.0 (7.2, 18.7)	10.7 (6.4, 19.2)	10.5 (5.7, 18.1)	0.029
	ICU LOS, days	5.0 (2.8, 9.2)	4.6 (2.6, 9.7)	4.8 (2.3, 8.3)	5.0 (2.9, 9.0)	5.2 (3.1, 10.1)	0.281
	In-hospital death, n (%)	609 (33.7)	113 (24.8)	128 (28.4)	163 (36.1)	205 (45.5)	<0.001
	ICU death, n (%)	468 (25.9)	70 (15.4)	95 (21.1)	126 (27.9)	177 (39.2)	<0.001
Other clinical outcomes							
	Sepsis	1236 (68.4)	263 (57.7)	283 (62.9)	315 (69.8)	375 (83.1)	<0.001
	AKI, n (%)	1024 (56.6)	217 (47.6)	237 (52.7)	262 (58.1)	308 (68.3)	<0.001

COPD, chronic obstructive pulmonary disease; SOFA, sequential organ failure assessment; OASIS, Oxford Acute Severity of Illness Score; APS-III, Acute Physiology Score III; MAP, mean arterial pressure; RR, respiratory rate; WBC, white blood cell; AST, aspartate aminotransferase; ALT, alanine aminotransferase; APRI, aspartate aminotransferase to platelet ratio index; BUN, blood urea nitrogen; PT, prothrombin time; APTT, activated partial thromboplastin time; INR, international normalized ratio; LOS, length of stay; ICU, intensive care unit; AKI, acute kidney injury.

### 3.2 APRI and the Primary Outcomes

**Fig. 2.**
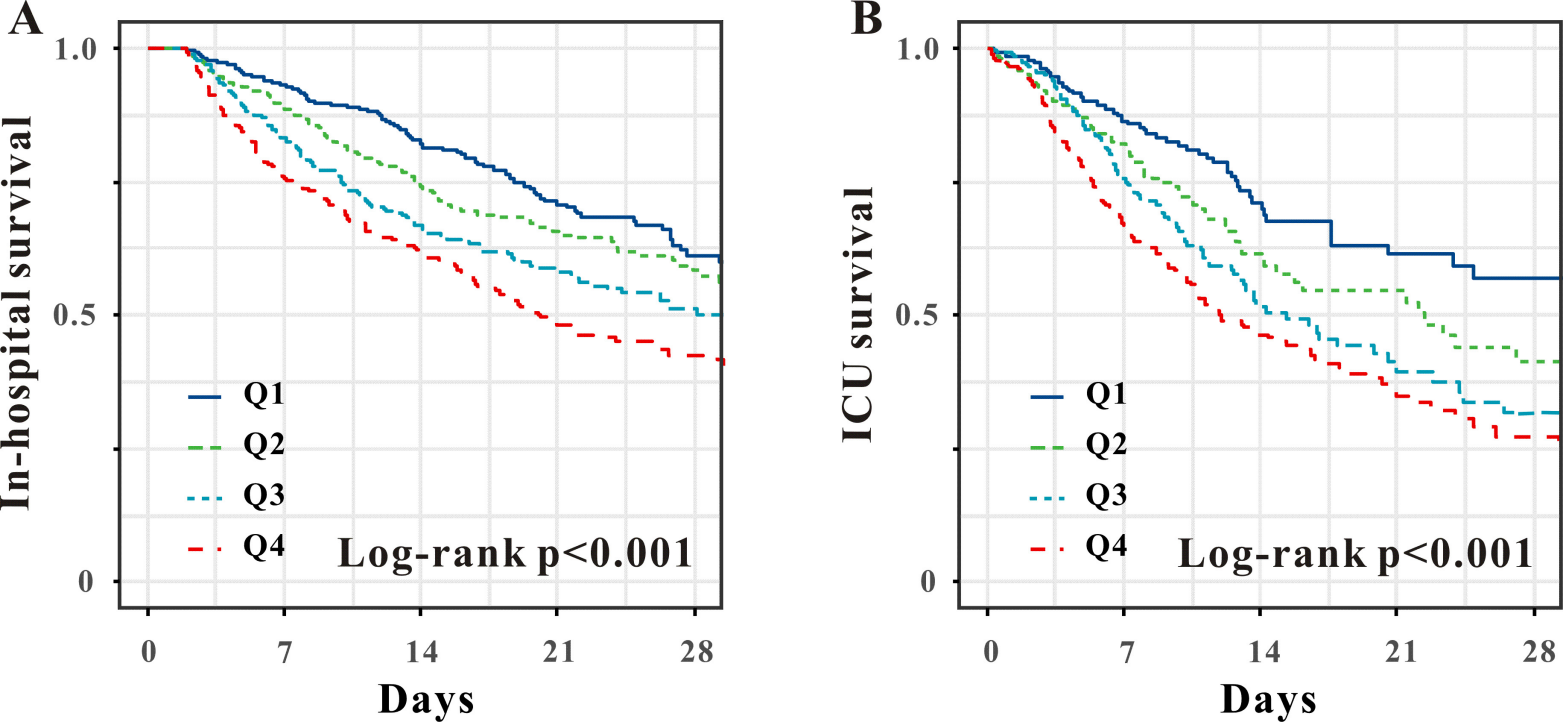
**Kaplan–Meier survival analysis curves of the APRI quartiles for in-hospital (A) and ICU mortality (B)**. APRI, aspartate aminotransferase to platelet ratio index; ICU, intensive care unit.

Multivariable Cox regression analysis revealed that APRI was independently associated with a heightened risk of death in-hospital (hazard ratio (HR) 1.005 [95% confidence interval (CI) 1.003–1.007]; *p* < 0.001) and in the ICU (HR 1.005 [95% CI 1.003–1.007]; *p* < 0.001; Table 2). The findings were additionally confirmed in the fully adjusted Model 3, which revealed that patients in the highest APRI quartiles exhibited the greatest risk of both in-hospital and ICU mortality (Table 2 and Fig. 2A,B).

**Table 2.** **The association between APRI and the primary outcomes**.

Exposure		Model 1		Model 2		Model 3	
		HR (95% CI)	*p*	HR (95% CI)	*p*	HR (95% CI)	*p*
In-hospital mortality							
	APRI as continuous	1.005 (1.003–1.007)	<0.001	1.005 (1.003–1.008)	<0.001	1.003 (1.001–1.005)	0.037
	Q1	Ref.		Ref.		Ref.	
	Q2	1.249 (0.970–1.609)	0.085	1.224 (0.950–1.577)	0.118	1.168 (0.903–1.512)	0.237
	Q3	1.656 (1.303–2.106)	<0.001	1.694 (1.330–2.157)	<0.001	1.443 (1.124–1.852)	0.004
	Q4	2.204 (1.751–2.773)	<0.001	2.407 (1.907–3.038)	<0.001	1.717 (1.327–2.221)	<0.001
	*p* for trend	<0.001		<0.001		<0.001	
ICU mortality							
	APRI as continuous	1.005 (1.003–1.007)	<0.001	1.006 (1.004–1.008)	<0.001	1.004 (1.001–1.007)	0.002
	Q1	Ref.		Ref.		Ref.	
	Q2	1.499 (1.101–2.042)	0.010	1.491 (1.094–2.032)	0.012	1.368 (0.995–1.881)	0.054
	Q3	1.885 (1.407–2.525)	<0.001	1.871 (1.394–2.512)	<0.001	1.640 (1.206–2.231)	0.002
	Q4	2.388 (1.811–3.150)	<0.001	2.627 (1.985–3.478)	<0.001	2.030 (1.498–2.753)	0.047
	*p* for trend	<0.001		<0.001		<0.001	

APRI, aspartate aminotransferase to platelet ratio index; ICU, intensive care unit; HR, hazard ratio; 95% CI, 95% confidence interval; Ref., reference. Model 1: unadjusted; Model 2: adjusted for age, gender, ethnicity, and comorbidity; Model 3: adjusted for Model 2 plus severity scores and laboratory results.

### 3.3 APRI and Other Clinical Outcomes

Multivariable logistic regression analysis also revealed that the APRI was independently correlated with an elevated risk of sepsis (odds ratio (OR) 1.106 [95% CI 1.070–1.144]; *p* < 0.001; Table 3) and AKI (OR 1.054 [95% CI 1.035–1.073]; *p* < 0.001; Table 3). These findings were additionally corroborated in the fully adjusted Model 3, revealing that patients in the highest APRI index quartile exhibited a significant association with an elevated risk of sepsis (OR 2.472 [95% CI 1.667–3.568]; *p* < 0.001; Table 3) and AKI (OR 1.85 [95% CI 1.305–2.624]; *p* < 0.001; Table 3) as compared to the lowest quartile. The incidence of sepsis and acute kidney injury exhibited a continuous increased trend with ascending APRI quartiles, with all trends *p* < 0.001 (Table 3).

**Table 3.** **The association between APRI and other clinical outcomes**.

Exposure		Model 1		Model 2		Model 3	
		OR (95% CI)	*p*	OR (95% CI)	*p*	OR (95% CI)	*p*
Sepsis							
	APRI as continuous	1.106 (1.070–1.144)	<0.001	1.107 (1.070–1.145)	<0.001	1.064 (1.031–1.098)	<0.001
	Q1	Ref.		Ref.		Ref.	
	Q2	1.370 (1.047–1.791)	0.022	1.379 (1.052–1.808)	0.020	1.229 (0.881–1.715)	0.224
	Q3	1.533 (1.169–2.010)	0.002	1.564 (1.189–2.057)	0.001	1.380 (1.006–1.894)	0.046
	Q4	3.621 (2.659–4.930)	<0.001	3.718 (2.717–5.088)	<0.001	2.472 (1.667–3.568)	<0.001
	*p* for trend	<0.001		<0.001		<0.001	
Acute kidney injury							
	APRI as continuous	1.054 (1.035–1.073)	<0.001	1.061 (1.041–1.081)	<0.001	1.041 (1.022–1.060)	<0.001
	Q1	Ref.		Ref.		Ref.	
	Q2	1.340 (1.032–1.740)	0.028	1.330 (1.016–1.740)	0.038	1.232 (0.912–1.663)	0.174
	Q3	1.395 (1.074–1.811)	0.013	1.493 (1.140–1.955)	0.004	1.264 (0.924–1.729)	0.143
	Q4	2.372 (1.810–3.109)	<0.001	2.742 (2.069–3.634)	<0.001	1.850 (1.305–2.624)	0.001
	*p* for trend	<0.001		<0.001		<0.001	

APRI, aspartate aminotransferase to platelet ratio index; OR, odds ratio; 95% CI, 95% confidence interval; Ref., reference. Model 1: unadjusted; Model 2: adjusted for age, gender, ethnicity, and comorbidity; Model 3: adjusted for Model 2 plus severity scores and laboratory results.

### 3.4 Subgroup Analysis

**Fig. 3.**
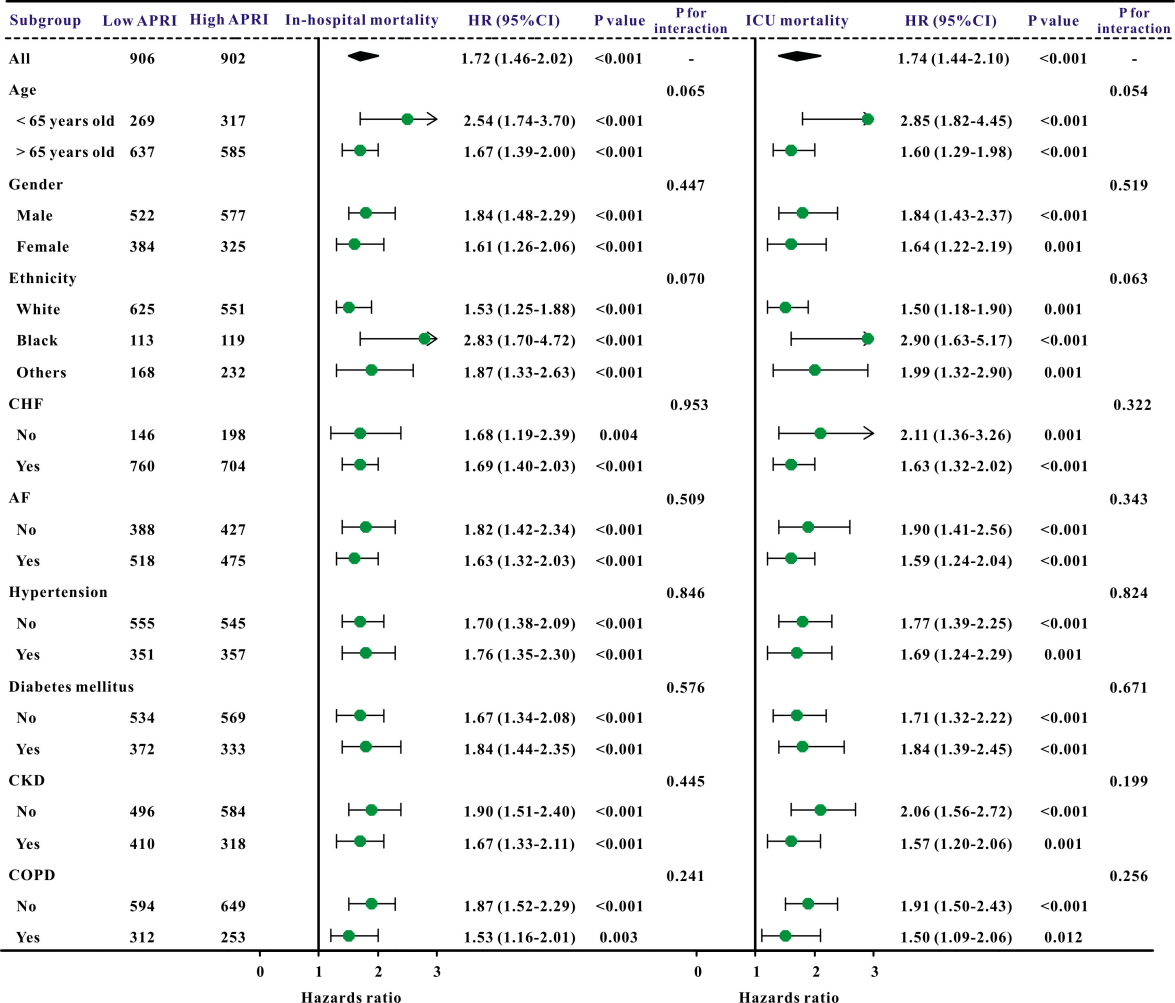
**A forest plot revealed the results of subgroup analysis for in-hospital mortality and ICU mortality based on the APRI value**. APRI, aspartate aminotransferase to platelet ratio index; ICU, intensive care unit; HR, hazard ratio; 95% CI, 95% confidence interval; COPD, chronic obstructive pulmonary disease; CHF, congestive heart failure; AF, atrial fibrillation; CKD, chronic kidney disease.

**Fig. 4.**
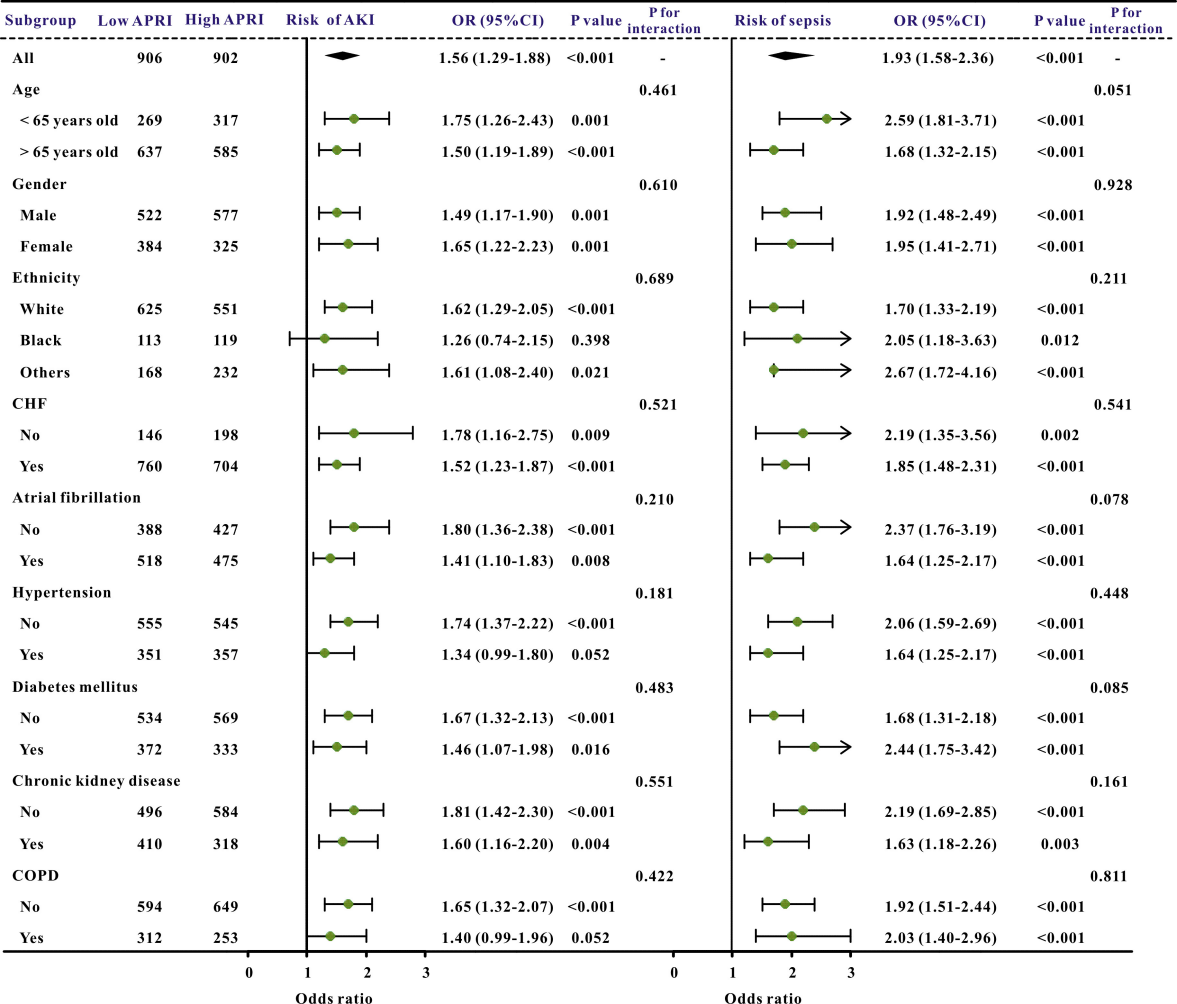
**The forest plot revealed the results of subgroup analysis for sepsis and acute kidney injury based on the APRI value**. APRI, aspartate aminotransferase to platelet ratio index; OR, odds ratio; 95% CI, 95% confidence interval; COPD, chronic obstructive pulmonary disease; AKI, acute kidney injury; CHF, congestive heart failure.

Additionally, subgroup studies based on specific features were conducted further to validate the association between the APRI and clinical outcomes. The APRI was markedly correlated with an increased risk of in-hospital mortality and ICU mortality across nearly all subgroups, in addition to the risk of AKI and sepsis (Figs. 3,4).

## 4. Discussion

To our knowledge, the present study is the first to uncover the potential role of employing the APRI in the prognosis prediction of critically ill CS patients. The current investigation demonstrated that the APRI independently correlated with ICU and in-hospital mortalities. Additionally, a higher risk of sepsis and AKI was linked to an elevated APRI. Moreover, this association remained even after considering various clinical and laboratory parameters.

Previous studies have explored several predictive tools to screen high-risk CS patients. The RESCUE score exhibited a promising instrument for predicting early mortality in patients with primary refractory CS and for decision-making [20]. The PRECISE score included fifteen predictors demonstrating high in-hospital mortality predictive performance in CS patients [21]. Moreover, the ACS-MCS score could also effectively stratify risk for all-cause mortality for CS patients [22]. However, although these scoring systems can effectively evaluate the prognosis of CS patients, they are complex for critical care physicians to assess the condition and predict outcomes. Therefore, it is necessary to explore additional clinically available biomarkers to predict the prognosis of CS patients.

The APRI represents a possible unobtrusive option to a liver biopsy for detecting hepatic fibrosis, which combines the AST level and platelet count, is easy to use, and does not require any special knowledge to interpret these two widely used serum indicators. Moreover, the APRI has been widely used to predict prognosis in different liver disease settings. Ashouri *et al*. [23] found that the APRI was significantly linked with liver failure in chemotherapy patients. Zhang *et al*. [24] indicated that the APRI is a potential index for the late recurrence of hepatocellular carcinoma after radiofrequency ablation. Lin *et al*. [25] performed a meta-analysis to investigate the connection between the APRI and fibrosis, concluding that the APRI can evaluate hepatitis C-related fibrosis with reasonable precision.

However, the predictive role of the APRI in the prognosis prediction of CS patients has yet to be explored; thus, our findings fill the gap in this area. Our data demonstrated that the APRI is a prognostic factor of poor outcomes in critically ill CS patients. Meanwhile, a higher APRI in patients indicated a higher risk of in-hospital mortality and ICU mortality alongside sepsis and AKI in CS patients admitted to the ICU. In previous studies, the APRI has been used to predict mortality [26, 27, 28]. The APRI has also been found to be effective in risk-stratifying patients with non-alcoholic fatty liver disease [26] and HIV-associated *Talaromyces marneffei* [27]. Maegawa *et al*. [28] demonstrated that the APRI was associated with perioperative mortality and overall survival after hepatectomy for hepatocellular carcinoma (HCC). However, the APRI does not always have a good predictive effect on mortality. Another study concluded that the APRI was not associated with post-surgical recurrence and mortality in cholangiocarcinoma patients who underwent surgical resection [29]. The present study expanded the predictive value of the APRI for mortality related to CS. Additionally, this study also found that the APRI was associated with sepsis except for in a few subgroups (black ethnicity and those with hypertension and COPD history) and AKI in CS patients admitted to the ICU.

However, as a widely-used liver fibrosis indicator, the underlying potential mechanisms for why it can be associated with the prognosis of CS patients remain largely unknown. Hence, investigating the correlation between liver fibrosis and cardiovascular disease is warranted due to the liver’s essential function in lipid and glucose metabolism and the common risk factors of hypertension, insulin resistance, and systemic inflammation that characterize both conditions [30, 31, 32]. A previous study indicated that an elevation in the APRI correlates with an augmented chance of mortality among the American population [33]. Indeed, the APRI has previously forecast mortality and the risk of cardiac-related deaths in patients with chronic cardiovascular disease [34]. Furthermore, the APRI is associated with the calcification of coronary arteries and its degree of severity in patients with ischemic cardiovascular illness [35]. A distinct investigation assessed the relationship between fibrous liver scores and thrombus or bleeding incidents in individuals with acute coronary syndrome. The results indicated that those with high APRI scores demonstrated a 1.57- to 3.73-fold increase in the incidence of crucial severe events after correction [36].

The APRI combined AST level and serum platelet level; higher APRI values were linked to poor outcomes in CS patients, which means higher AST and/or lower platelet levels in these patients. CS is defined by diminished heart output, resulting in decreased blood circulation and oxygenation. In CS, hepatic impairment frequently occurs because the heart’s rhythm could prove inadequate to satisfy the nutrient needs of liver cells. Moreover, liver dysfunction during the early stages was observed in 25% of those diagnosed with CS and correlated separately with fatalities. Transcription factor levels may serve as an alternative indicator for the circulatory system in severe heart failure, and an increase in transaminase levels within a single day is also related to reduced existence [37]. CS patients may occasionally develop liver injury, such as congestive liver disease and hepatic hypoperfusion, as a result of decreased cardiac output [38]. Transaminase levels typically rise dramatically and sharply in hypoxic hepatitis, which is indicative of liver cell destruction. Therefore, in absolute terms, transaminase levels can serve as a biomarker for hemodynamic reserve and are linked to low in-hospital mortality rates [39]. In the case of CS, abnormal liver function test results are observed in both cases of decreased perfusion and venous congestion. Hence, liver dysfunction is common in CS patients, and higher AST levels could predict poor outcomes in these patients. Moreover, low platelets are closely related to bleeding risk, and previous study indicated low platelets predicted poor outcomes in different disease settings [40]. Another study also found that low platelet is a prognostic factor for mortality in ICU [41]. A previous study found that platelet decrease during ICU hospitalization was robustly linked to evaluated death in CS patients [42]. Since platelets are the main mediator of the immune system, immune dysregulation caused by thrombocytopenia may increase inflammatory responses, increase the risk of sepsis, and significantly increase the risk of death in CS patients. This is also a possible reason for the higher incidence of sepsis in thrombocytopenia patients [43, 44]. Thrombocytopenia at CS presentation was associated with sepsis and worse clinical findings, including liver and renal functions. The authors speculate that it is caused by the dysregulation of inflammatory response in patients with thrombocytopenia, as platelets often act as inflammatory mediators and participate in neurohormones, which play a key part in the pathophysiology of CS [45]. This study also found that thrombocytopenia was linked to a greater inflammatory response; the leukocyte increased with APRI quartiles. Together, liver damage caused by insufficient liver perfusion, thrombocytopenia, immune response, and inflammatory response are potential mechanisms; however, further studies need to clarify the underlying mechanisms.

Several limitations of the present study should be acknowledged, the first of which was its retrospective design; subsequent prospective trials are needed to confirm whether the APRI in this population is linked to an increased risk for specific clinical outcomes. Second, extensive studies with larger sample sizes investigating dynamic changes in the APRI could provide additional evidence to support our conclusions because all baseline characteristics were gathered within 24 hours after ICU admission, and there is a lack of data regarding dynamic changes in the APRI during the hospital stay. Third, although this study adjusted for several variables, such as comorbidities and severity scores, the APRI seems to be able to predict clinical outcomes independently. However, some liver and hematological diseases may affect the AST and platelet values. Due to database limitations, we could not obtain data on whether patients had comorbidities such as liver and hematological diseases that may affect the APRI values. Thus, further confirmation of the results of this study is needed in the future.

## 5. Conclusions

The present study explored the utility of the APRI, an easy, non-invasive, and immediately measured marker for predicting clinical outcomes in critically ill patients diagnosed with CS. The results of this study provide supportive evidence that an increased APRI is independently associated with poor clinical outcomes. However, additional prospective studies are needed to validate these findings.

## Availability of Data and Materials

The datasets used during the current study are available from the corresponding author upon reasonable request.
